# Magnetic resonance imaging of umbilical cord stem cells labeled with superparamagnetic iron oxide nanoparticles: effects of labelling and transplantation parameters

**DOI:** 10.1038/s41598-020-70291-9

**Published:** 2020-08-13

**Authors:** Akiko Ohki, Shigeyoshi Saito, Kazuki Fukuchi

**Affiliations:** 1 Department of Medical Physics and Engineering, Division of Health Sciences, Osaka University Graduate School of Medicine, Suita, Osaka 560-0871 Japan; 2 Department of Biomedical Imaging, National Cardiovascular and Cerebral Research Center, Suita, Osaka 565-8565 Japan

**Keywords:** Imaging, Preclinical research

## Abstract

Cell tracking with magnetic resonance imaging (MRI) is important for evaluating the biodistribution of transplanted cells. Umbilical cord-derived mesenchymal stem cells (UC-MSCs) have emerged as a promising therapeutic tool in regenerative medicine. We examined the UC-MSCs labeled with superparamagnetic (SPIO) and ultrasmall superparamagnetic iron oxide (USPIO) in terms of cell functioning and imaging efficiency in vitro and in vivo. The UC-MSCs were co-incubated with SPIO or USPIO at a concentration of 50 or 100 µg/mL of label. Viability and proliferation were assessed by Trypan blue dye exclusion and MTT assay, respectively. Differentiation (chondrogenesis, osteogenesis, and adipogenesis) was induced to examine the impact of labelling on stemness. For in vitro experiments, we used 7-T MRI to assess the T_2_ values of phantoms containing various concentrations of cell suspensions. For in vivo experiments, nine neonatal rats were divided into the control, SPIO, and USPIO groups. The UC-MSCs were injected directly into the rat brains. MRI images were obtained immediately and at 7 and 14 days post injection. The UC-MSCs were successfully labeled with SPIO and USPIO after 24 h of incubation. Cell viability was not changed by labelling. Nevertheless, labelling with 100 µg/mL USPIO led to a significant decrease in proliferation. The capacity for differentiation into cartilage was influenced by 100 µg/mL of SPIO. MRI showed that labeled cells exhibited clear hypointense signals, unlike unlabeled control cells. In the USPIO-labeled cells, a significant (*P* < 0.05) decrease in T_2_ values (= improved contrast) was observed when compared with the controls and between phantoms containing the fewest and the most cells (0.5 × 10^6^ versus 2.0 × 10^6^ cells/mL). In vivo, the labeled cells were discernible on T_2_-weighted images at days 0, 7, and 14. The presence of SPIO and USPIO particles at day 14 was confirmed by Prussian blue staining. Microscopy also suggested that the regions occupied by the particles were not as large as the corresponding hypointense areas observed on MRI. Both labels were readily taken up by the UC-MSCs and identified well on MRI. While SPIO and USPIO provide improved results in MRI studies, care must be taken while labelling cells with high concentrations of these agents.

## Introduction

Mesenchymal stem cells (MSCs), a kind of somatic stem cells, are isolated from the mesenchymal tissues, such as fat, bone, and cartilage. MSCs can differentiate into mesodermal cells^1^, endodermal^2^, and ectodermal cells^3^. Because of their regenerative and immunoregulatory capacities^4^, MSCs are expected to be applied for the therapy of various diseases, such as osteogenesis imperfecta, ischemic stroke, and cardiac infarction.

Bone marrow is the most common source of MSCs. However, collecting MSCs from the bone marrow is highly invasive, and the maximal life span of the cells is affected by the donor’s age^5^. Currently, an umbilical cord tissue called Wharton’s jelly is attracting attention, as a promising source of stem cells because these tissues are normally discarded at birth and MSCs can be readily isolated from them. Umbilical cord-derived MSCs (UC-MSCs) are an ideal therapeutic tool due to their high proliferation capacity^6,7^, immune-privileged status^8,9^, and absence of ethical issues. Currently, although the need for UC-MSC applications to regenerative medicine is growing the available technologies have not been fully studied.

To ensure the successful stem-cell treatment, it is crucial to monitor the transplanted cells for the appropriate biodistribution and migration. The current approaches for cell tracking include imaging with magnetic resonance imaging (MRI), positron emission tomography, single photon emission computed tomography, and fluorescence imaging^10^. MRI is widely used imaging modality for cell tracking, which can noninvasively observe the transplanted cells when they are labeled with contrast agents, such as superparamagnetic (SPIO) or ultrasmall superparamagnetic iron oxide (USPIO)^10^. Imaging with MRI contrast agents is more preferable than other approaches because of its high resolution, safety, and no effects of radiotracer.

The iron oxide nanoparticles used in MRI studies are divided into commercially synthesized^11^ or laboratory-synthesized particles^12,13^. The synthesized particles lack in reproducibility and have difficulty in controlling and monitoring^14^. Moreover, it is suggested that the synthesis technique should be further improved^15^. Therefore, the commercially synthesized nanoparticles are preferable because of the immediate availability and proven safety. Resovist is a commonly used SPIO procedure for the detection and characterization of focal liver lesions^16,17^, and is currently available in a limited number of countries^18^. Molday ION Rhodamine B (MIRB) is a novel USPIO designed for cell labelling and tracking. It requires no transfection agent^19^ and can be applied to multiple cell types^20^. These contrast agents create local magnetic field inhomogeneities that decrease the signal on T_2_- and T_2_*-weighted magnetic resonance (MR) images^21,22^.

In this study, we compared the influence of SPIO and USPIO on UC-MSC function, labelling efficacy, and in-vivo distribution on 7-T MRI.

## Results

### Prussian blue staining

**Figure 1:**
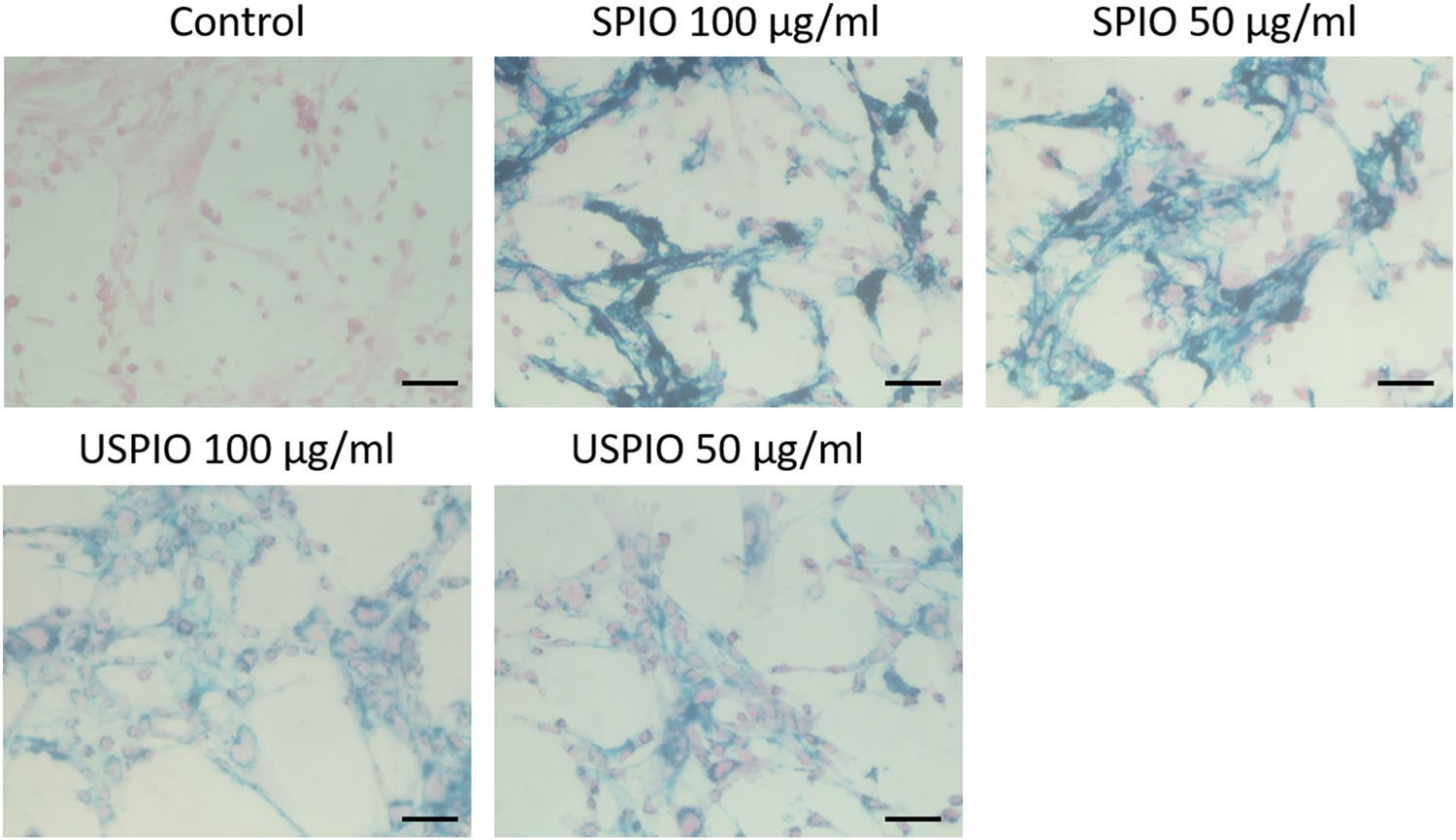
Prussian blue (blue)/eosin (pink) staining of the unlabeled and iron-oxide-labeled UC-MSCs. The cells were cultured with 50 or 100 µg/mL of SPIO or USPIO particles. Scale bars, 50 µm. *UCMSCs* umbilical cord-derived mesenchymal stem cells, *SPIO* superparamagnetic iron oxide, *USPIO* ultrasmall superparamagnetic iron oxide.

After 24 h, the UC-MSCs were labeled with SPIO or USPIO, as confirmed by microscopic examination (Fig. 1). SPIO and USPIO particles appeared as blue-stained granules in the cytoplasm. UC-MSCs took up SPIO and USPIO particles in increasing amounts with increasing the concentrations. No morphological changes were observed in any group.

### Cell viability and proliferation

**Figure 2:**
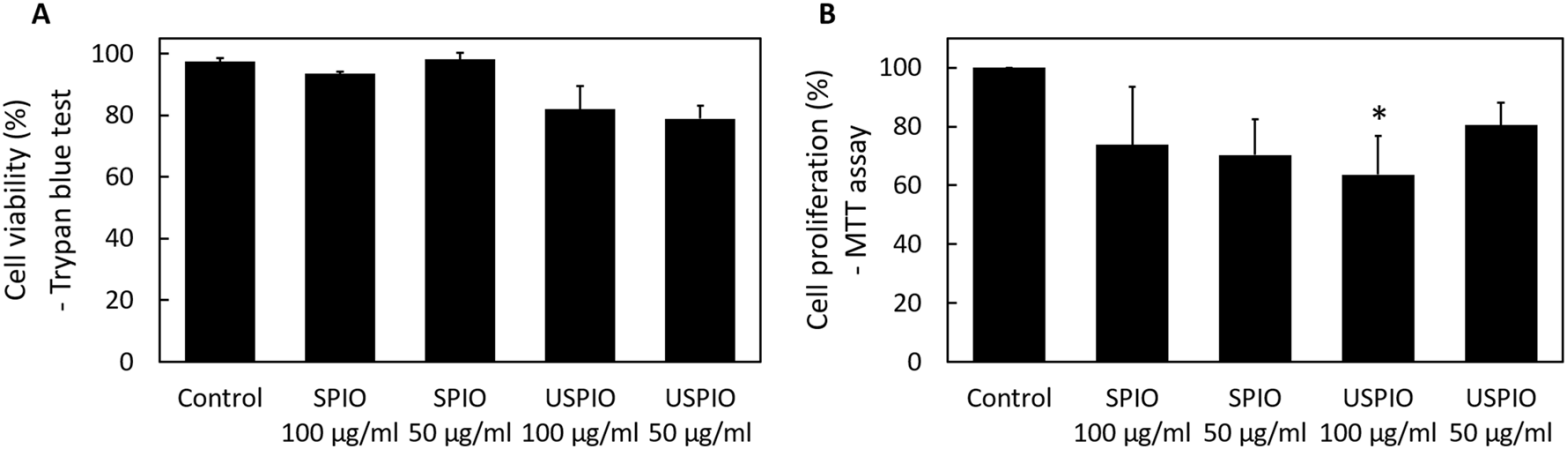
The effects of labelling on UCMSCs (**A**) Cell viability as determined by the Trypan blue exclusion assay. Viability is calculated as follows: percent viable cells = 100 × (number of viable cells per mL of aliquot)/(total number of cells per mL of aliquot). No significant impairment is observed. (**B**) Cell proliferation, as evaluated by the MTT assay (MTT: 3‐[4,5‐dimethylthiazol‐2‐yl]‐2,5‐diphenyltetrazolium bromide). Proliferation as a percent is calculated as follows: 100 × (optical density of “treated” well − blank)/(optical density of “control” well − blank) (i.e., the optical density measurements are scaled to those of the untreated control cells). A significant impairment is observed at 100 μg/mL of USPIO. *P < 0.05 compared with control. *UCMSCs* umbilical cord-derived mesenchymal stem cells, *SPIO* superparamagnetic iron oxide, *USPIO* ultrasmall superparamagnetic iron oxide.

The Trypan blue exclusion test revealed no significant decreases in the viability of the labeled compared with the corresponding of the unlabeled cells (Fig. 2A). However, the MTT assay demonstrated that in cells treated with 100 µg/mL of USPIO, proliferation was significantly lower than that of the unlabeled cells (Fig. 2B; *P < 0.05).

### Differentiation capacity

**Figure 3:**
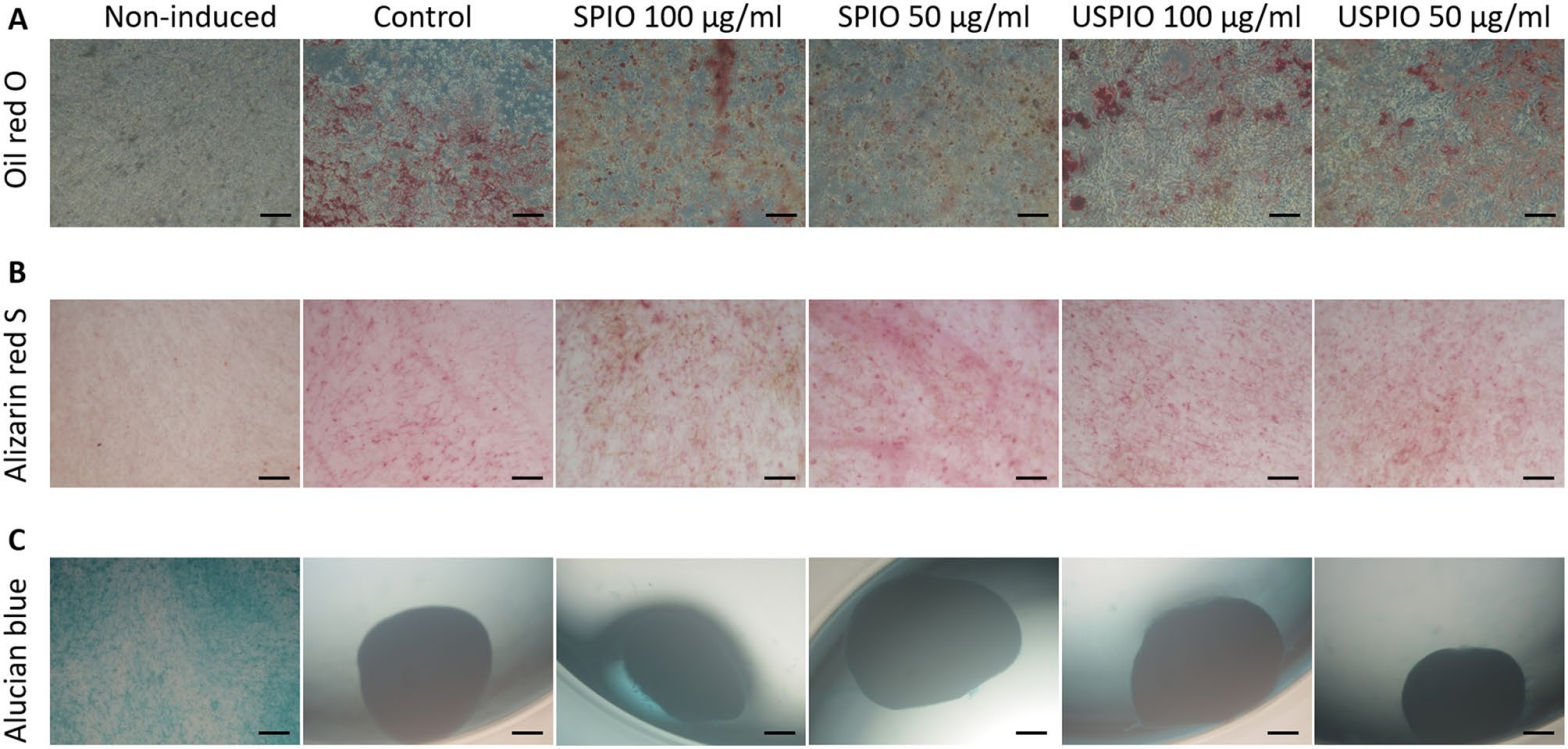
The effect of the labelling protocol on UCMSC differentiation is presented. (**A**) Adipogenic differentiation is detected by Oil red O staining; all normal. (**B**) Osteogenic differentiation is detected by Alizarin red S staining; all normal. (**C**) Chondrogenic differentiation is detected by Alcian blue staining; a small impairment is observed at 100 μg/mL of SPIO. Oil red O and Alizarin red S: scale bar, 100 µm; Alcian blue: scale bars, 200 µm. *UCMSCs* umbilical cord-derived mesenchymal stem cells, *SPIO* superparamagnetic iron oxide, *USPIO* ultrasmall superparamagnetic iron oxide.

Induction of adipogenesis was demonstrated by the presence of lipid vesicles stained with Oil red O in all induced groups (Fig. 3A). The osteogenic differentiation assay revealed that all induced groups expressed calcium deposition, as detected by Alizarin red S (Fig. 3B). Chondrogenic differentiation was also observed in all induced groups, which formed cartilaginous spheroids that stained dark blue with Alcian blue (Fig. 3C). However, after labelling with 100 µg/mL of SPIO, the cells did not form complete spheroids and partly stained light blue, which resembled the staining behavior of the non-induced cells (Fig. 3C).

### In vitro* MRI*

**Figure 4:**
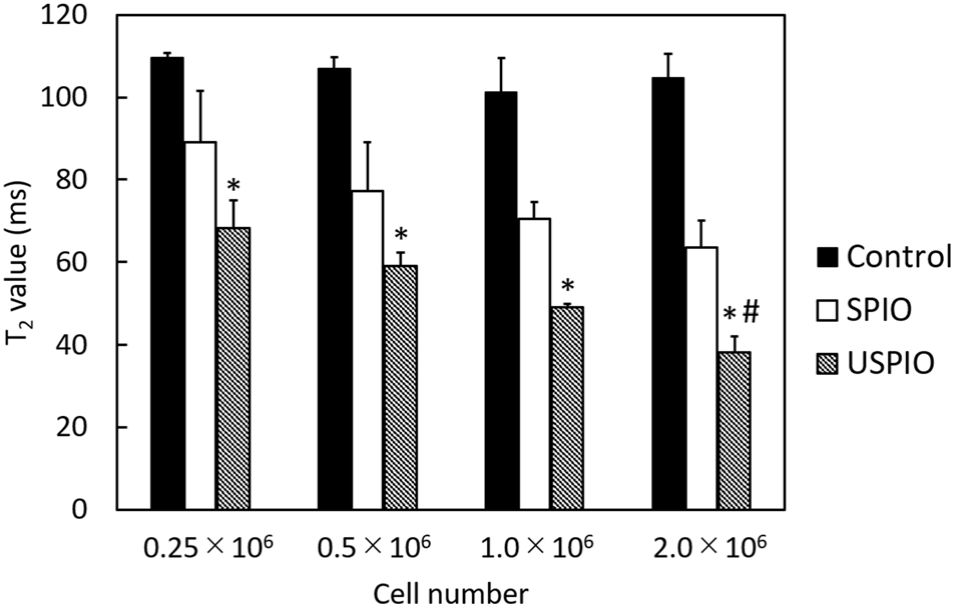
T_2_ values (contrast) in MRI of phantoms containing different concentrations of unlabeled and labeled UC-MSCs. USPIO shows a dose-dependent effect. *P < 0.05, compared with control group; ^#^*P* < 0.05, compared with concentration = 0.25 × 10^6^ cells/mL in the USPIO series. *UCMSCs* umbilical cord-derived mesenchymal stem cells, *SPIO* superparamagnetic iron oxide, *USPIO* ultrasmall superparamagnetic iron oxide, *MRI* magnetic resonance imaging.

The measured T_2_ values of different concentrations of labeled and unlabeled cells are shown in Fig. 4. The cells labeled with SPIO or USPIO demonstrated lower T_2_ values than the unlabeled cells. A significant decrease in T_2_ value was observed in the USPIO-labeled cells at concentrations of 0.25 × 10^6^, 0.5 × 10^6^, 1.0 × 10^6^, and 2.0 × 10^6^ cells/mL compared with that of the control (Fig. 4; *P < 0.05). When the cells were treated with USPIO, the phantom with 0.25 × 10^6^ cells/mL showed a significant difference in T_2_ value compared with 2.0 × 10^6^ cells/mL (Fig. 4; ^#^*P* < 0.05).

### In vivo* MRI*

**Figure 5:**
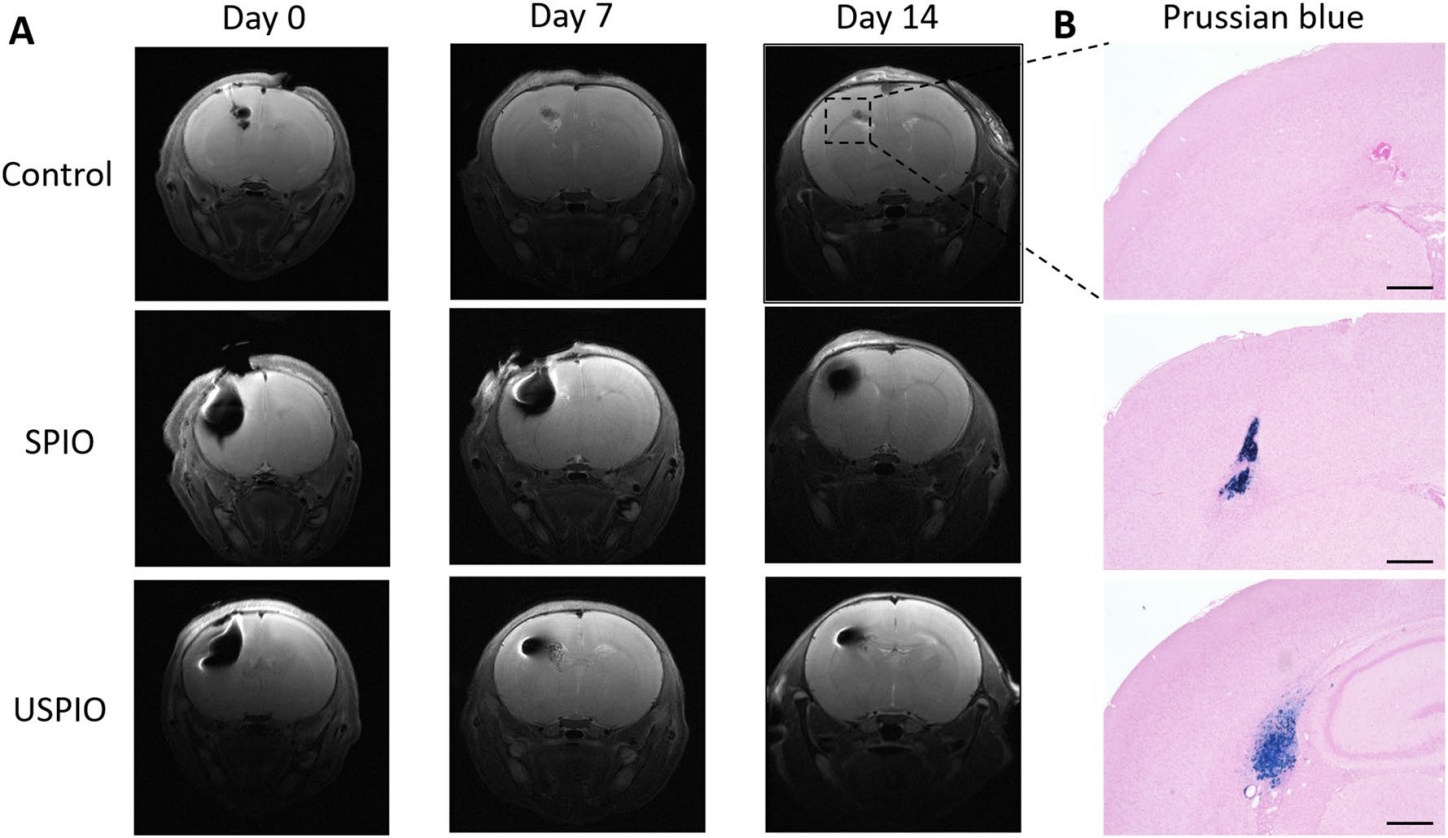
In vivo results: (**A**) T_2_-weighted MRI of the control (upper, *n* = 1), SPIO (middle, *n* = 4), and USPIO (lower, *n* = 4) animal groups at 0, 7, and 14 days after injection of UC-MSCs. (**B**) Micrographs of the low-signal-intensity regions observed on MRI at day 14. SPIO and USPIO deposits are demonstrated in the left hemisphere by Prussian-blue staining. Scale bars, 500 μm. *UCMSCs* umbilical cord-derived mesenchymal stem cells, *SPIO* superparamagnetic iron oxide, *USPIO* ultrasmall superparamagnetic iron oxide, *MRI* magnetic resonance imaging.

There was one death in the USPIO group at day 14, during MRI scanning. T_2_W images obtained at days 0, 7, and 14 are shown in Fig. 5A. On the day of the injection, the control rats showed a small hypointense area with a line extending downward from the cortex, indicating a needle track. The hypointense areas in the control animals were barely recognizable at days 7 and 14. In all eight rats in the SPIO and USPIO groups, the implanted cells were clearly observed at day 0, displaying hypointense signals on T_2_W images. Although the hypointense areas were slightly reduced by day 7, the areas were discernible in all rats with labeled cells and were similar to those at day 14, except for one rat in the USPIO group. The hypointense region size appeared to be generally smaller in the USPIO than in the SPIO group at 7 and 14 days post injection. The presence of SPIO and USPIO particles οn day 14 was confirmed by Prussian blue staining (Fig. 5B). SPIO tended to present more densely in the brain tissue compared to USPIO. The blue-stained regions were not as large as the corresponding of low signal intensity observed on the MR images.

## Discussion

In this study, we evaluated the characteristics of UC-MSCs labeled with SPIO (Resovist) or USPIO (MIRB) by in vitro and in vivo experiments, to investigate the suitability of these agents for cell tracking. We demonstrated that simple co-incubation was sufficient for labelling with Resovist and MIRB, and that the labeled cells could be detected by MRI.

Cellular uptake depends on the physicochemical properties of nanoparticles. There are some mechanisms of the incorporation of nanoparticles, such as passive diffusion, and receptor, clatharin, and caveoline mediated endocytosis^23^. It is assumed that the nanoparticles are taken up into cells generally through clathrin- mediated or caveolin- mediated pathways due to their shape and size^24^. Transfection agents, such as Lipofectamine^25^, poly-l-lysine^26^, and protamine^27^, are often used to achieve intracellular uptake of contrast agents. However, transfection agents generally have cytotoxicity^28,29^ and most of them are not clinically approved^30^. Therefore, labelling cells without the use of transfection agents would seem to be advantageous.

It has been reported that the diameter of particles influences their cellular uptake; USPIO at 20 nm or larger particles at 900 nm give lower cellular loadings than Resovist at 62 nm^31^. Mailänder et al. showed that Resovist was more effectively taken up into bone marrow-derived MSCs than Feridex, a SPIO–dextran composite, and suggested that the difference in biopolymers was responsible for the results^32^. MIRB was also successfully incorporated into the UC-MSCs in the present study. MIRB is a USPIO contrast agent designed for cell labelling, and has a mean particle diameter of 35 nm and a positive zeta potential of + 31 mV at physiological pH (available from the manufacture’s data). In contrast, Resovist has a zeta potential of − 20 mV at pH 7.0^33^. It is known that surface charge plays an important role in cellular uptake. Positively charged particles are more readily taken up by cells than negative or neutral particles^34,35^, as cationic chemical groups disturb the cell membrane potential^36^ or induce reconstruction of the lipid bi-layers^37^. Thus, in this study, the particle diameter, surface coating, charge, or a combination of these factors, likely produced efficient uptake, resulting in clear MR signals.

SPIO and USPIO did not affect viability or proliferation except for 100 µg/mL of MIRB in the proliferation assay. It has been reported that iron oxide nanoparticles generate intracellular reactive oxygen species resulting in oxidative stress and cellular toxicity^38^. Addicott et al. demonstrated by flow cytometry that co-incubation of cells with concentrations of MIRB above 30 µg/mL for 20 h reduced viability, which reached a value of 78.8% at 100 µg/mL^39^. They also showed that at 10 and 20 µg/mL, the proliferation capacity, as assessed by fold expansion, was similar to that of the unlabeled cells^39^. These suggest that toxicity estimates may depend on the measuring method used and that adding high concentrations of label seems to negatively impact cell survival.

The ability to produce multiple mesenchymal cell lineages, such as osteocytes, chondrocytes, and adipocytes, is crucial for cells to be defined as MSCs. We showed that 100 µg/mL of SPIO (Resovist) slightly affected the formation of cartilage cells. Henning et al. reported that ferucarbotran (Resovist) dose-dependently inhibited chondrogenesis in MSCs, but the inhibition became insignificant after decreasing the incubation time to 4 h^40^. Chen et al. demonstrated that Resovist at a high concentration (300 µg/mL) for 1 h reduced alkaline phosphatase expression, a marker of osteoblasts, and prevented osteogenic differentiation by promoting cell migration and activating specific molecules^41^. They reported that the inhibition of osteogenic differentiation is caused dose-dependently, and free iron in the medium plays an important role in the inhibition. Therefore, the differences in concentration and incubation time are likely to be related with the difference between the results of our study and previous reports. Particularly, in cell-based therapy, these factors can have adverse effects on stemness and, thus, in therapeutic effect.

In the phantom studies, the T_2_ values of USPIO-labeled cells were significantly lower than those of the unlabeled cells. Additionally, a significant effect of cell concentration was observed in the USPIO group. The small size of USPIO particles controls the T_2_/T_1_ relaxation time, resulting in low T_2_ values^42^. Our results indicated that the amount of the USPIO-labeled UC-MSCs could be estimated from the T_2_ values, which can facilitate the monitoring of cell-based therapies. In vivo, the signal loss was observed until day 14, indicating that the SPIO- and USPIO-labeled cells are useful for longitudinal cell observation. At 14 days after transplantation, the SPIO and USPIO particles were densely retained, as shown by histological staining. Moreover, the blue-stained regions were smaller than the corresponding regions of signal loss observed on the T_2_W images. This suggested that the regions detected by 7-T MRI extended beyond the physical location of the nanoparticles. It is known that at high field strength, iron oxide nanoparticles disturb the magnetic field with strong R_2_ and R_2_* effects^43,44^. Furthermore, iron oxide particles accumulate in the cytoplasm, and the cells form clusters when in suspension^20^, which causes a much stronger R_2_* than R_2_ effect^45^. These characteristics are advantageous for observing cells, but are liable to cause overestimation of the number of transplanted cells, due to the blooming effect or susceptibility artifacts. The properties and parameters of the nanoparticles should be cautiously considered to achieve optimal image-based diagnosis.

We acknowledge that this study had several limitations. First, cell fate in vivo was not evaluated in detail. It was not clear from our results whether the gradual signal loss was due to the decreased numbers of viable cells. Histological and histochemical analyses in combination with MRI at various time points are essential for the interpretation of the SPIO or USPIO-induced signal voids. Second, the therapeutic efficacy of the labeled UC-MSCs was not investigated. As the UC-MSCs can be used safely and with minimal ethical complications, they are expected to become the preferred therapeutic stem cell type, especially in neonatal disorders, such as hypoxic-ischemic encephalopathy^46–48^. Further studies are necessary to investigate the possibilities of using UC-MSCs in cell-based treatments.

In summary, we conclude that the UC-MSCs labeled with Resovist or MIRB are suitable for longitudinal MRI studies. MIRB, a notable USPIO, was especially suitable, because it allowed quantitative cell tracking. Care should be taken in adding SPIO or USPIO at high concentrations. The results of this study support future applications of the UC-MSCs with cell tracking by MRI and contrast agents.

## Materials and methods

### Approval statement

All experimental protocols using animals were performed in accordance with the Guidelines for Animal Experiments at National Cardiovascular and Cerebral Research Center. All experiments and procedures were approved by the Institutional Animal Care and Use Committee of National Cardiovascular and Cerebral Research Center (approval number, 18019).

### Cell culture and labelling

Human UC-MSCs (PromoCell, Heidelberg, Germany) were cultured in MSC growth medium (PromoCell). The cells were seeded at a density of 0.09 × 10^5^ cells/cm^2^ in cell-culture dishes or wells and cultured at 37 °C under 5% CO_2_ to 70–80% confluency. All experiments were performed between passages 3 and 6. The UC-MSCs were labeled for 24 h with a SPIO (Resovist; Fujifilm Toyama Chemical Co., Ltd., Tokyo, Japan) or USPIO (MIRB; BioPAL, Worcester, MA, USA), at final concentrations of 50 or 100 µg/mL, then, washed three times with phosphate-buffered saline (PBS) (Nakalai Tesque Inc., Kyoto, Japan).

### Prussian blue staining

After washing in PBS, the cells were fixed with 4% paraformaldehyde in PBS (PFA) for 30 min, stained for 20 min with 2% potassium ferrocyanide in 2% hydrochloric acid, and counterstained with 1% eosin (FUJIFILM Wako Pure Chemical Co., Osaka, Japan). The SPIO and USPIO particles were observed with a Zeiss Axiovert 25 inverted microscope (Zeiss, Oberkochen, Germany).

### Cell viability assay

Cell viability was assessed by Trypan blue dye exclusion assay. The cells were exposed to Trypan blue dye (FUJIFILM Wako Pure Chemical), and 10 µL of cell suspension was applied to a hemocytometer. The viable (unstained) and nonviable (stained) cells were counted to calculate the percentage of viable cells as follows:

$$ \begin{aligned} {\text{Viability }}\left( \% \right) & = 100 \times {{\left( {\text{number of viable cells per mL of aliquot}} \right)} \mathord{\left/ {\vphantom {{\left( {\text{number of viable cells per mL of aliquot}} \right)} {\left( {\text{total number of cells}} \right.}}} \right. \kern-\nulldelimiterspace} {\left( {\text{total number of cells}} \right.}} \\ & \quad \left. {\text{per mL of aliquot}} \right). \\ \end{aligned} $$

### Proliferation assay

Proliferation was assessed by MTT assay (MTT: 3‐[4,5‐dimethylthiazol‐2‐yl]‐2,5‐diphenyltetrazolium bromide). The MSCs were grown in 96-well plates. Ten microliters of MTT solution (Nakalai) were added to each well and incubated for 4 h at 37 °C under 5% CO_2_. Then, 100 µL of solubilization solution (Nakalai) was added to each well to dissolve the MTT formazan. Then, the absorbance was measured at 570 nm using a microplate reader (Varioskan Flash; Thermo Scientific, MA, USA). The percent proliferation capacity was calculated as follows:

$$ \begin{aligned} & 100 \times {{\left( {{\text{optical density of treated well}}{-}{\text{optical density of blank}}} \right)} \mathord{\left/ {\vphantom {{\left( {{\text{optical density of treated well}}{-}{\text{optical density of blank}}} \right)} {\left( {\text{optical density of}} \right.}}} \right. \kern-\nulldelimiterspace} {\left( {\text{optical density of}} \right.}} \\ & \left. {{\text{control well}}{-}{\text{optical density of blank}}} \right),\left( {\text{the optical density measurements were scaled}} \right. \\ & \left. {\text{to those of the untreated control cells}} \right). \\ \end{aligned} $$

### Differentiation assay

The capacity for differentiation into the adipose, bone, and cartilage cells was evaluated for the labeled and unlabeled cells. MSC growth medium was used in the remaining well as a negative control. To induce adipogenic differentiation, the cells were seeded at a density of 0.1 × 10^5^ cells/cm^2^ into six-well plates coated with fibronectin (10 µg/mL) and cultured in adipogenic differentiation medium (PromoCell) for 14 days. The medium was changed every 3 days. Then, the cells were fixed with 4% PFA for 30 min and stained with Oil red O (Nakalai). To induce osteogenic differentiation, the cells were seeded at a density of 0.1 × 10^5^ cells/cm^2^ into six-well plates coated with fibronectin (10 µg/mL) and cultured in osteogenic differentiation medium (PromoCell), which was changed twice a week for 2 weeks. Then, the cells were stained with Alizarin red S (Nakalai) followed by fixation in 4% PFA for 30 min. To induce chondrogenic differentiation, the cells were seeded at a density of 30 × 10^4^ cells/cm^2^ into 96-well U-bottom culture plates without any pre-coating procedure. The cells were incubated in chondrogenic differentiation medium (Biological Industries, Beith Haemek, Israel) with two medium changes per week. After 21 days of incubation, the cells were fixed with 4% PFA for 60 min and stained with Alcian blue (FUJIFILM Wako Pure Chemical). All stained cells were photographed by light microscopy.

### Magnetic resonance imaging

The UC-MSCs were treated with SPIO or USPIO at a concentration of 100 µg/mL. The rinsed cells were detached using Accutase (PromoCell) and resuspended in PBS. The experiments to acquire MR images of phantom samples and animal brains were performed using a horizontal 7-T scanner (BioSpec 70/30 USR; Bruker Biospin, Ettlingen, Germany) equipped with a transmit-receive volume coil having an inner diameter of 72 mm and a four-channel mouse-brain coil.

### In vitro

To prepare the phantoms, centrifuge pellets of MSCs were embedded in agarose gel at 0.25 × 10^6^, 0.5 × 10^6^, 1.0 × 10^6^, and 2.0 × 10^6^ cells/mL. MR images were obtained using a multi-slice, multi-echo, spin echo with the following parameters: repetition time (TR) = 2000; echo time (TE) = 8 ms; slice thickness = 1.0 mm; field-of-view = 60 × 60 mm^2^; matrix size = 128 × 128; slice number = 1; number of averages = 1; slice orientation = coronal; and scan time = 4 min, 16 s. T_2_ values were calculated within a 0.09-cm^2^ region in the center of the phantom image.

### In vivo

The following three groups were studied: SPIO (*n* = 4), USPIO (*n* = 4), and control (*n* = 1) groups. The surgeries were performed under general anesthesia with isoflurane: 3.0% for induction and 2.0% for maintenance. Nine 8-day-old Wistar rats (three males and six females; Japan SLC Inc., Shizuoka, Japan) were habituated to the rearing environment before the experiment. A microinjector was inserted into the left side of the skull to a depth of 3 mm. Intracerebral transplantation of a 3-µL suspension of 3 × 10^5^ cells was performed in a stereotaxic device at an infusion rate of 0.5 µL/min. To obtain MR images, the rats were positioned in a stereotaxic frame with mouth and ear bars to prevent movements during acquisition. The body temperature of the rats was maintained at 36.5 °C with regulated water flow and continuously monitored using a physiological monitoring system (SA Instruments Inc., Stony Brook, USA). T_2_-weighted images (T_2_WIs) were acquired with the RARE (RARE: rapid acquisition with relaxation enhancement) with the following parameters: TR = 2,500; TE = 33 ms; RARE factor = 8; slice thickness = 0.8 mm; field-of-view = 19.2 × 19.2 mm^2^; matrix size = 256 × 256; slice number = 20; number of averages = 4; slice orientation = trans-axial; and scan time = 5 min, 20 s. The MR images were acquired immediately and at 7 and 14 days post injection. After the MRI session at day 14, the animals were sacrificed and the brain sections prepared and stained with Prussian blue.

### Statistical analysis

Data are presented as means ± standard deviations (*n* = 3). Differences were compared using the Kruskal–Wallis test followed by Dunn's post-test to evaluate cell viability, proliferation, and the T_2_ values of the phantoms. All analyses were performed using Prism 5, Version 8 (GraphPad Software, CA, USA, https://www.graphpad.com/). *P* < 0.05 was considered statistically significant.
